# Identification and mapping of a new recessive dwarfing gene *dw9* on the 6RL rye chromosome and its phenotypic effects

**DOI:** 10.1371/journal.pone.0229564

**Published:** 2020-03-02

**Authors:** Agnieszka Grądzielewska, Paweł Milczarski, Katarzyna Molik, Edyta Pawłowska

**Affiliations:** 1 Institute of Plant Genetics, Breeding and Biotechnology, University of Life Sciences, Lublin, Poland; 2 Department of Plant Genetics, Breeding and Biotechnology, West Pomeranian University of Technology, Szczecin, Poland; Institute of Genetics and Developmental Biology Chinese Academy of Sciences, CHINA

## Abstract

The introduction of high-yielding semi-dwarf varieties of wheat into cultivation has led to a "green revolution." This has required intensive research into various sources of dwarfism in wheat. However, there has been very little advancement in research on dwarfing genes in rye in comparison to wheat or barley. So far, three dominant dwarfing genes (*Ddw*1, *Ddw*3, and *Ddw*4) and three recessive genes (*ct*1, *ct*2, and *np*) have been characterized and precisely mapped in rye. There is no complete catalog of dwarfing genes available in rye. This paper presents an identification of the source of dwarfism and preliminary characterization of the new recessive gene *dw*9 from the BK-1 line. The gene was mapped on the long arm of the 6R chromosome and belongs to the GA-insensitive group. The initial characterization of the influence of this gene on morphological traits shows that it significantly affects the decrease of yielding trait parameters. A full evaluation can be performed after detailed breeding studies.

## Introduction

Rye (*Secale cereale* L.) is a crop species from the tribe *Triticeae* (*Poaceae*). It is an important crop, especially for the region referred to as “rye belt,” which stretches from Northern Germany to Central and Northern Russia. In Germany, Poland, Russia, and former Soviet Union countries, this national crop is used mainly for bread baking and feed production. Compared with wheat, barley, or triticale, rye is still a very tall crop. Rye populations cultivated in Poland reach up to 150–160 cm in height, and hybrids are only a little smaller at 130–140 cm.

Together with the plant’s morphology (slender culm with long internodes and long spike), the considerable height is undesirable because weather conditions such as strong wind, storms, and rain make rye very vulnerable to lodging. Lodging is a very unfavorable occurrence that decreases yield level and grain quality [1]. This problem is often resolved using chemical plant growth regulators (PGRs), but the high costs and the pollution of the soil and grain with chemicals have prompted a search for other solutions [1,2].

Progress in the understanding of crop genetics and the identification of dwarfing genes has allowed their transfer to cultivars, resulting in a decrease of plant height. As a result, in some crops like wheat or barley, the lodging problem has been resolved without chemical protection. In the case of rye, dwarfing genes that efficiently decrease height without losses of yield are unknown. Until now, only four dominant dwarfing genes have been identified in rye: *Ddw*1 [3], *Ddw*2 [4], and recently, *Ddw* 3 [5] and *Ddw* 4 [6]. Attempts at introducing dwarfing genes to cultivars were carried in only the case of *Ddw*1 [4,7]. Unfortunately, in rye, the *Ddw*1 gene decreases yield, so it is not used yet in breeding. Interestingly, *Ddw*1 was successfully introduced to triticale cultivars, and in Poland, it has been the main dwarfing gene used by triticale breeders.

There is much information about the dominant dwarfing genes of rye *Ddw*1 [8], *Ddw*3 [5] and *Ddw*4 [6], but little is known about recessive ones. Several recessive dwarfing genes have been identified in rye. Some of them have been assigned to chromosomes and were localized on all seven rye (R) chromosomes (1R-7R). Only three of these genes were assigned to chromosomes arms: *ct*1 (7RL) [9], *ct*2 (5RL) [10], and *np* (*nana postratum*) (4RL) [15]. In the case of other gene, closer localization is unknown.

The nomenclature of the recessive genes is inconsistent. About half of them were named as “*dw*,” and the small group of three genes was designated as “*ct*” (*compactum*) genes (*ct*1—*ct*3) because of their dense spike morphology [9,10] [15]. The others have different and inconsistent nomenclature. It is possible that some of the genes are identical, especially those from 4R or 5R, because as many as three and four genes have been assigned to these chromosomes, respectively. Their authentication would be possible only after conducting a wide range of allelic complementation tests, but it seems impossible because a significant majority of recessive dwarfing gene sources are unavailable.

One of the useful methods for classifying dwarfing genes is the evaluation of their reaction to exogenously applied gibberellic acid (GA). This test allows dwarfing genes to be divided into two groups: one with dwarf genotypes that regain their normal height after GA application (sensitive genes) and those that show no reaction or a reduced reaction (insensitive genes) [11]. All four dominant dwarfing genes of rye were shown to be GA-sensitive [5,6,12]. Among recessives, *dw*1 [13], *dw*2 [14], *ct*3 [15], *np* [16], and *ds*1 [17] were classified as GA-sensitive, and *dw*6 [18], *ct*1 [10], *ct*2 [9] and *ds*2 [17] were shown to be GA-insensitive.

The second method is to use marker techniques that enable precise chromosome localization of the dwarfing gene on the genetic map. One of the possible technologies is the DArTseq^™^ method. This platform uses genome complexity reduction and the next-generation sequencing (NGS) of short DNA fragments on the Illumina platform [19,20]. The DArTseq^™^ technique has been applied for genotyping by sequencing [20–22], linkage mapping, QTL identification [23,24], GWAS (genome-wide association studies), MAS (marker-assisted selection), and GS (genomic selection) [25–27].

The aim of this study was to identify the recessive dwarfing gene of rye in the BK-1 inbred line, which was derived from *Bashirskaja karlikovaja* Russian source. We assigned this gene to rye chromosome, determined its reaction to GA_3_ and present the basic characteristics of its phenotypic effect.

## Materials and methods

### Plant material

The maternal inbred line 541 was obtained in West Pomeranian University of Technology, Szczecin. This self-fertile line has a tall phenotype and has been reproduced by self-pollination for more than 20 years. The detailed characteristics of the line were described by Milczarski *et al*. [28]. *Bashirskaja karlikovaja*, a Russian source of a recessive dwarfing gene, was kindly provided by the N.I. Vavilov All-Russian Scientific Research Institute of Plant Industry, St. Petersburg, Russia. The paternal BK-1 line was derived in the University of Life Sciences in Lublin by two cycles of self-pollination. The BK-1 line is characterised by at least 2 times higher tillering capacity than cultivated varieties or lines and flexed, semi-erect stem with high number of short internodes. This specific, not upright growth form results from the fact that from a given node successive internodes grow at an angle to the previous one. The nodes are significantly thicker than the diameter of the stem. The spikes are short and flat, filled with very small grains of low weight. In addition, one stem may have supernumerary ears growing from a lower node. The leaves of the BK-1 line are proportionally shorter and narrower in comparison to other rye varieties and lines.

The two parental lines were hybridized in 2012, and in the next season, 2 tall F_1_ plants (code: BK2 and BK3) were self-pollinated. In 2013, F_2_ populations were sown in Czesławice in the Experimental Farm of the University of Life Sciences in Lublin. The height of 331 plants of these populations was measured to verify the expected 3:1 ratio of segregation. The distribution of dwarfism in the F_2_ population was confirmed by observation of the F_3_ progeny (7–20 plants per line). If the number of plants assessed in generation F_3_ was less than 7, such a genotype was not classified into any of the groups (dwarf, tall, segregating), unless both tall and dwarf plants were visible. The hypothesis of the monogenic inheritance of plant height in F_2_ and F_3_ progenies was verified using the χ^2^ test.

The parents and F_2_ population were assessed for their morphological traits: plant height, length, and thickness of the second internode from the bottom, main spike length, and number and weight of kernels in the main spike. The measurements of the parental plants were performed in 2014–2016. The BK-1 line was also crossed with Dańkowskie Amber (Danko Ltd.) to test the gene effect and introduce the *dw*9 to a modern population variety. A basic characterization of these newly developed plant materials was conducted to assess six morphological traits, including thousand-kernel weight (TKW). The statistical analyses of the mean values, standard deviation, correlation coefficients, and significant differences were conducted using Statistica 13.3 software (TIBCO Software Inc.).

### Genetic mapping

Genomic DNA was extracted from young, healthy leaves of F_2_ plants and parental individuals using a GenElute^™^ Plant Genomic DNA Miniprep Kit (Sigma). DNA from 94 randomly chosen F_2_ plants and parents was used for DArTSeq genotyping (Diversity Arrays Technique Pty Ltd). The results obtained from phenotype segregations and the DArTSeq scores were used to construct a genetic map of the rye chromosome carrying the new recessive dwarfing gene. DNA from the F_2_ mapping population and parents was used for DArTseq genotyping (Diversity Arrays Technique Pty Ltd). Linkage and localization of the identified markers with the dwarfing gene were performed with Kosambi mapping function using JoinMap 5.0 software [29].

In the final analysis, only a linkage group with dwarfing gene segregation was constructed with an LOD ratio of 17. The linkage groups were thereafter compared to the RIL-S (population 541 × 2020) consensus genetic map of rye [30]. An original map showing the relationships to the reference map was generated using MapChart 2.0 software [31]. The chromosome location was verified using data from the reference sequence of the *Secale cereale* Lo7_v2 database [32]. For this purpose, scaffolds with a description of the chromosome 6R were extracted from the Sc_Lo7_v2 library. DArTSeq sequences were mapped to reference scaffolds (Sc_Lo7_v2_6R) and filtered out, and the chromosome position has been verified. All scaffolds to which DArTSeq markers have been mapped were annotated as well using a custom "*nt*" library. The DArTseq sequence mapping to reference scaffolds and an annotation analysis of selected scaffolds were performed using CLC Genomic Workbench 11.0 (QIAGEN Ltd.).

### GA_3_-seedling test

The GA_3_-seedling test was performed according to Worland [33] with minor modifications. Seeds of tall parental line 541 and the semi-dwarf BK-1 line were germinated on moistened filter paper. The seeds were kept at 4˚C for 48 h to synchronize germination and then kept at 20˚C for 3 days. Seedlings of both lines were then transferred into plastic boxes containing moistened perlite, and a standardized nutrient solution supplemented with 5 ppm of GA_3_ was applied every day until the seedlings reached the 2–3 leaf stage. The second group of plants of both lines was treated with a nutrient solution without GA_3_ (control group).

Plants were planted in three replications. At 14 days after the initiation of germination, the length of the seedlings was measured from the base to the top. Statistical analyses were performed to calculate significant differences between the means of groups using a t-Student’s test in Statistica 13.3 software (TIBCO Software Inc.).

## Results

### Genetic analysis of plant height in 541 × BK-1 crosses

**Fig 1:**
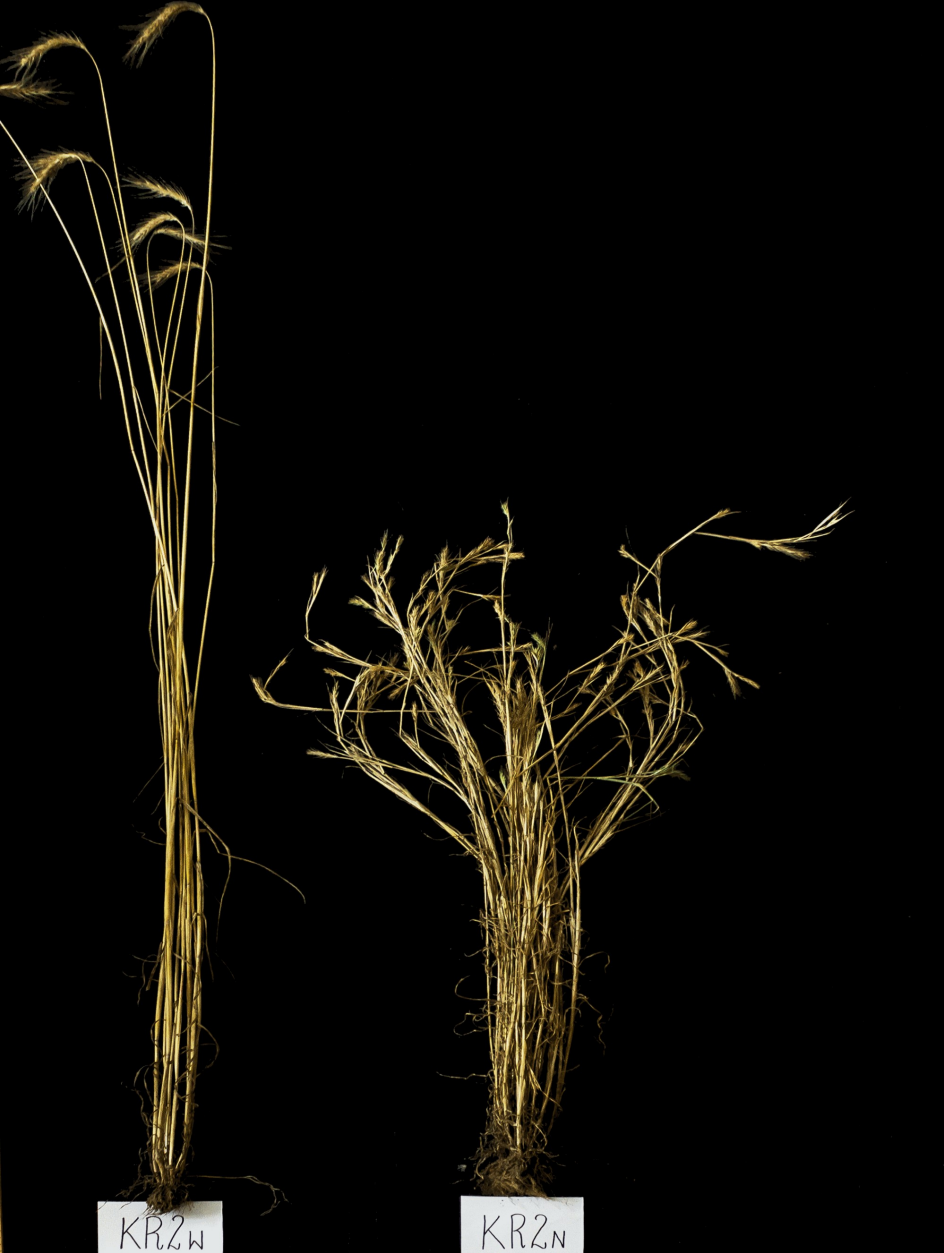
Tall and dwarf plants from BK mapping population. The tall phenotype corresponds to 541, and dwarf to BK-1 phenotype.

The phenotyping of plants from two parental lines showed significant differences between the BK-1 dwarf line and the 541 line of normal height (Table 1, Fig 1, S1 Table). The mean height of the dwarf line was 96.9 cm, and the plant height reduction determined by the dwarf gene present in the BK-1 line was slightly above 28% compared to the 541 line. The dwarf line had a low inbreeding level (S_2_) and a very low reproduction rate. All morphological features other than the number of stem internodes were significantly lower in the BK-1 mutant than in the maternal line. Especially dramatic were the differences in the number (75%) and weight of kernels per spike (90%). Additionally, the number of internodes occurring in line 541 was significantly different (almost three times smaller than in line BK-1). This is a characteristic of the morphology of the BK-1 mutant, which has a large number of stem internodes.

**Table 1:** Morphological traits of the mapping population parental lines (541 and BK-1).

Year		Plant height* [cm]		Length of 2-nd internode* [cm]		Thickness of 2-nd internode* [mm][		Spike length* [cm]		Number of kernels per spike*		Weight of kernels per spike*		Number of internodes*	
		541	BK-1	541	BK-1	541	BK-1	541	BK-1	541	BK-1	541	BK-1	541	BK-1
2014	Mean	137.15	101	19.40	7.10	4.53	2.38	11.24	5.65	49.90	13.2	1.15	0.10	5.7	15.0
	SD	4.63	7.15	1.61	2.28	0.32	0.32	0.68	0.88	7.05	1.75	0.26	0.01	0.46	2.57
2015	Mean	135.20	94.8	18.80	7.85	3.88	2.15	11.99	6.25	50.60	13.6	1.16	0.09	5.7	16.0
	SD	9.94	9.83	1.47	2.11	0.40	0.5	1.15	0.48	6.55	3.37	0.18	0.04	0.46	2.64
2016	Mean	132.90	94.9	19.26	7.05	4.68	1.95	10.70	5.52	54.20	11.05	1.74	0.11	5.6	13.7
	SD	5.80	4.99	3.59	1.74	0.58	0.29	0.69	0.75	15.14	2.00	0.71	0.02	0.49	1.55
**2014–2016**	**Mean**	**135.08**	**96.9**	**19.15**	**7.33**	**4.36**	**2.16**	**11.31**	**5.81**	**51.57**	**12.62**	**1.35**	**0.10**	**5.66**	**14.9**
	**SD**	**6.79**	**7.32**	**2.22**	**2.04**	**0.43**	**0.37**	**0.84**	**0.70**	**9.58**	**2.37**	**0.38**	**0.02**	**0.48**	**2.54**

*traits significantly different at p<0.01

**Fig 2:**
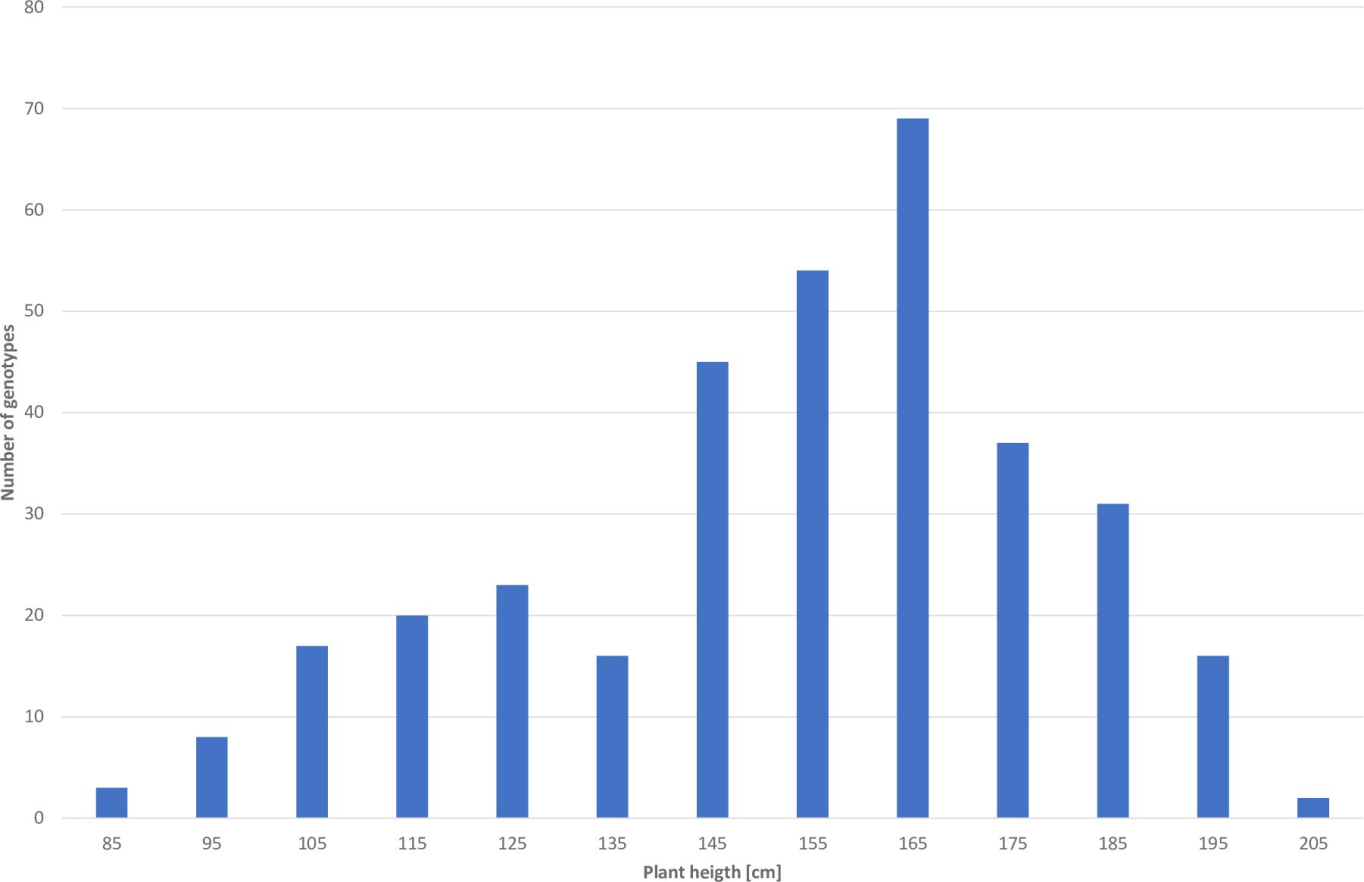
Distribution of plant height in F_2_ population of the 541 × BK-1 cross.

The height of the F_1_ generation was 150 cm, which suggested total domination of the normal growth gene over the corresponding recessive BK-1 allele. The range of plant height (75–206 cm) in the F_2_ generation of 331 plants exceeded the range of the parental lines, which suggests environmental or genetic background effects, including transgression (Table 2, Fig 2, S1 Table). The height distribution of F_2_ progeny was bimodal, which shows that this generation consists of two subpopulations: a more numerous long straw subpopulation (132–206 cm) with an average height of 160.7 cm and a smaller one representing short straw plants (75–130 cm) with an average height of 109 cm.

**Table 2:** Segregation of dwarf vs. tall phenotypes among plants of F_2_ and F_3_ progeny.

Generation	Range of plant height [cm]	Dominant homozygotes	Heterozygotes	Reccessive homozygotes	Expected segregation ratio	χ^2^
F_2_	75–206	257		74	3 : 1	1.23 (n.s.)*
F_3_	69–178	84	132	60	1 : 2 : 1	4.69 (n.s.)*

*n.s.- not significant deviation from the Mendelian ratio as assessed by χ^2^ test at p<0.05

The lowest height attributed to the phenotypes of high plants was 130 cm because no dwarf plants were observed in the F_3_ progeny with this value, and genotypes of this height were segregated. The analysis of F_3_ progeny allowed us to distinguish homozygotes from heterozygotes within the dwarf gene range. The coefficient of segregation of short and high stem phenotypes in F_2_ was confirmed by the observation of F_3_ offspring and was consistent with a ratio of 3:1. This suggests a monogenic inheritance of plant height in the 541 × BK-1 cross (Table 2, S1 Table).

### Mapping of the dwarfing gene *dw*9

Out of 53,433 DArTseq markers, 10,979 were selected in the Mendelian model within the F_2_ mapping population for the 541 × BK-1 cross. The gene segregation of the dwarfism gene was added to the pool of genetic markers, and several linking groups were obtained. One group included 437 markers to which the dwarfing gene was linked. Using the data from the 541 × 2020 reference map, the selected markers located on chromosome 6R of the reference map were verified.

**Fig 3:**
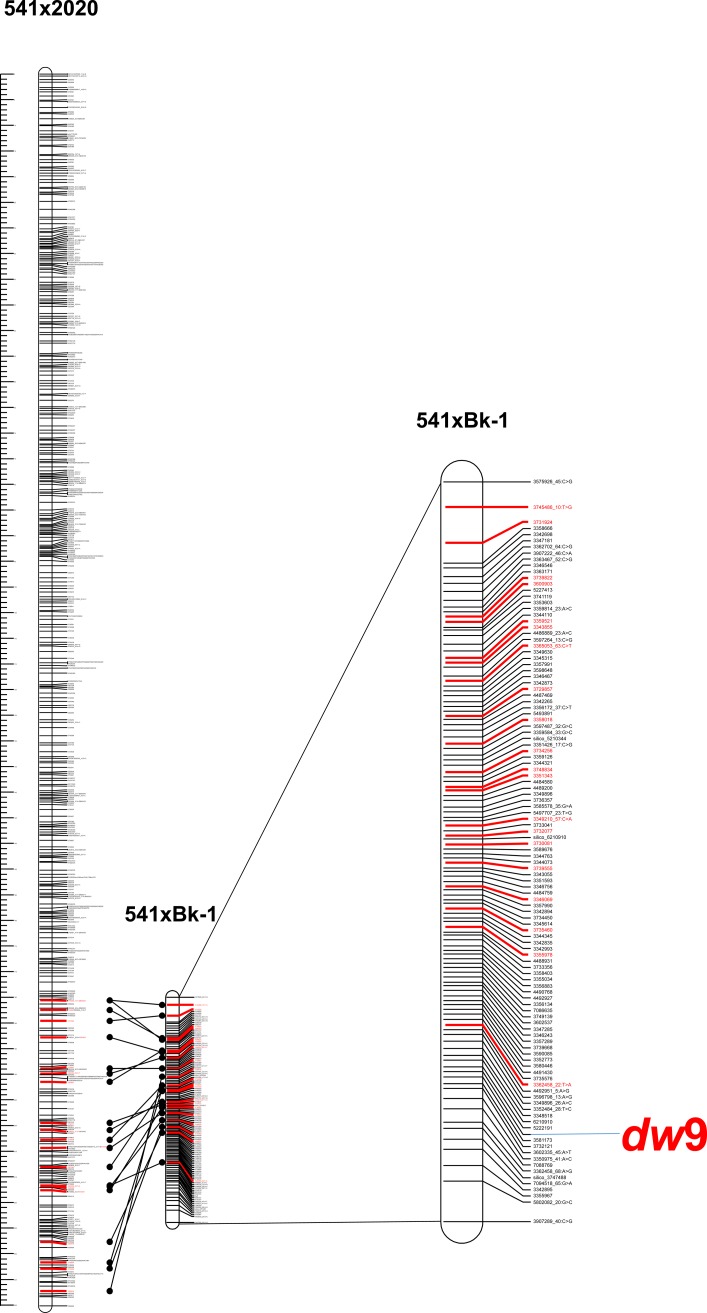
Chromosomal localisation of *dw*9 gene. The reference map of 6R chromosome (541 × 2020) comes from Milczarski *et*.*al*. [30].

Precise map analysis allowed to place the dwarfing gene on the distal part of the long arm of the 6R chromosome in position 214.98 cM (Fig 3, S2 Table). A high-density map for this segment was obtained using 112 markers and compared with the position of common markers on the reference map of the 6R chromosome. The genetic distances between the markers of the linkage group containing the dwarfing gene have been recalculated to their respective positions on the reference map (population 541×2020). The markers order of the 541 x BK-1 mapping population is in good agreement with the reference map, although numerous but minor marker position rearrangements are also observed. The gene is located in a cluster of 67 markers surrounding it at a distance of +/- 1 cM, which can be used to mark it and make a marker-assisted selection. The DArTSeq markers with the numbers 5222191 and 3581173 flanking the dwarfing gene position are very strongly linked to it over distances of 0.05 and 0.06 cM respectively. It was observed that this difference is related to genotype B2, which during the analysis of dwarfism segregation was determined as recessive homozygot (dwarf genotype), while the same genotype analyzed with markers 5222191 and 3581173 shows a heterozygous type or one corresponding to homozygotes specific for tall plants.

This is the first time that a map of the dwarfing gene location has been presented here, so the new gene has been assigned the symbol *dw*9.

To validate the chromosome location, 112 DArTSeq markers were mapped to the reference library of the Sc_Lo7_v2_scaffolds rye genome sequence associated with only the 6R chromosome. From the total pool of all scaffolds in the library, the 81 491 scaffolds were filtered out, and the Sc_Lo7_v2_scaffolds_6R sublibrary was created. In this group, part of the scaffolds has a chromosome position (cM), and part is assigned to only the 6R chromosome (without position). Of the total pool of 112 markers DArTSeq, 54 had compatible sequences with 45 scaffolds of the Sc_Lo7_v2_scaffolds_6R library (S3 Table).

The data on the chromosome location of scaffolds revealed that those with marked chromosome positions were in the range of 150.47 to 179.34 cM (that is, precisely in the distal segment of the 6R chromosome). Scaffolds selected from the reference library were annotated to search for a potential candidate gene for a newly discovered dwarfing gene (S3 Table). The data analysis showed that none of the scaffolds contain sequences that may be functionally related to the dwarfism caused by the *dw*9 gene. One of the most strongly linked DArTSeq (3581173) markers with the *dw*9 gene showed 94% of sequence similarity to the corresponding scaffold sequence Lo7_v2_scaffold_617669. The further analysis revealed its annotation to the hypothetical proteins TRIUR3_29124 from Triticum urartu (Accession no. EMS51228), which does not show potential relationship with dwarfism.

### Sensitivity of *dw*9 to GA_3_

The BK-1 line showed a very low degree of sensitivity to exogenous GA_3_ in the seedling phase (Table 3, S1 Table). The line reaction was statistically insignificant but much smaller than the one in line 541. Thus, it can be concluded that the *dw*9 gene in line BK-1 belongs to the group of GA-insensitive genes.

**Table 3:** Reaction of seedlings of parental lines to exogenously applied GA_3_.

Genotype	Mean length of seedlings [cm]		GA_3_/control	Level of significance
	control (H_2_O)	GA_3_ treated		
541	16.53	23.80	144.4%	p < 0.05
BK-1	8.72	9.51	109.1%	ns

### Evaluation of the effect of *dw*9 on agronomic traits of rye

The noticeable, statistically significant differences between parental lines BK-1 and 541 in relation to the analyzed traits translated into the correlations between them observed in the F_2_ generation of the hybrid (Table 4). All six traits were positively and statistically significantly correlated with each other, and the smallest correlations were observed between the length of the second internode and the number and weight of kernels per spike (r <0.5). The number and weight of kernels per spike were most strongly correlated with each other (r = 0.94). Unfortunately, the highly significant correlations of plant height with spike length and the number and weight of kernels per spike found in the progeny of F_2_ suggest that these three traits may be adversely affected by the dwarfing gene *dw*9 (Table 4). This is a rather negative result for the use of the *dw*9 gene in breeding.

**Table 4:** Correlation coefficients between studied traits in 541 × BK-1 F_2_ mapping population.

Traits	Plant heigth [cm]	Length of 2-nd internode [cm]	Thickness of 2-nd internode [mm]	Spike length [cm]	Number of kernels per spike	Weight of kernels per spike [g]
Plant heigth [cm]	-					
Length of 2-nd internode [cm]	**0.59**	-				
Thickness of 2-nd internode [mm]	**0.72**	**0.57**	-			
Spike length [cm]	**0.68**	**0.54**	**0.82**	-		
Number of kernels per spike	**0.59**	**0.45**	**0.69**	**0.73**	-	
Weight of kernels per spike [g]	**0.61**	**0.48**	**0.69**	**0.71**	**0.94**	-

Significant correlation coefficients at: p<0.01 are in bold

False positive or false negative interpretations may result from using only inbred lines to evaluate the effect of the dwarfism gene on rye genotypes and evaluating the effects based on only the analysis of correlations in the F_2_ population between such lines. To verify the effects of the dwarfing gene *dw*9, line BK-1 was crossed with the population variety Dańkowskie Amber. Table 5 shows preliminary and only one-year results illustrating the influence of *dw*9 on agronomic values of cv. Dańkowskie Amber. The F_1_ Dańkowskie Amber × BK-1 hybrid revealed significant heterosis of plant height (14%), 2 internode elongation (16%), and significant reduction of TKW (18.5%). Average values for other traits were nominally lower, but the differences were not statistically significant. However, the results confirmed the significant and negative influence of the *dw*9 gene on the most important yield-forming traits, especially on the weight of 1000 grains, observed in parental forms.

**Table 5:** Characteristics of morphological traits in *cv*. Dańkowskie Amber and F_1_ Dańkowskie Amber x BK-1 cross.

	Plant height* [cm]		Length of 2-nd internode* [cm]		Thickness of 2-nd internode [mm[		Spike length [cm]		Number of kernels per spike		Weight of kernels per spike [g]		TKW* [g]	
	Mean	SD	Mean	SD	Mean	SD	Mean	SD	Mean	SD	Mean	SD	Mean	SD
Dańkowskie Amber	132.10	7.21	12.00	1.18	3.80	0.03	10.12	0.91	43.10	7.89	1.87	0.54	33.63	4.03
Dańkowskie Amber x BK-1 (F_1_)	151.0	11.86	14.6	2.05	3.4	0.40	9.4	1.30	44.6	10.43	1.7	0.49	27.4	3.21

*- traits showing significant difference between Dańkowskie Amber and F_1_ (Dańkowskie Amber × BK-1), t–Student test at p<0.05

## Discussion

The catalogue of known dwarfing genes in rye was presented previously by Börner *et al*. [11]. In the list of recessive dwarfing genes in rye, only one described by Melz [18] as *dw*7, was reported on the 6R chromosome. Apart from assigning 6R to the chromosome based on trisomic set analysis, there is no additional information allowing for the precise determination of its location. The reaction of this gene to exogenously applied GA_3_ is also unknown. Therefore, it is difficult to determine the relationship between the *dw*7 gene described by Melz [18] and the one characterized in this paper. Answering this question will be possible after crossing the BK-1 with the I-1006 line, the source of the *dw*7 gene.

The newly discovered *dw*9 gene was precisely located on the high-density genetic map of 6R chromosome in the distal region of the long arm of this chromosome. A comparison of the closely linked marker segment with the dwarfing gene showed that it corresponds to the 6RL location of the reference map 541 × 2020 population in position between 180.0 and 223.9 cM [30]. This position was also confirmed by the scaffold analysis of the reference rye genome base of known chromosome location [32]. This double verification leaves no doubt about the chromosome location of the *dw*9 gene especially as compatible positions of the population map markers 541 × BK-1 and scaffolds are observed. Additionally, there was an insignificant reaction of the dwarf line to exogenous GA_3_, which positions the gene as being insensitive to gibberellin. It can be concluded that this gene should represent GA signaling genes, but further research is needed to confirm this.

An additional annotation analysis was performed for the Sc_Lo7_v2_scaffolds_6R library scaffold sequences to which DArTSeq markers were mapped, which did not show that any of the scaffold sequences had functionally related dwarfism sequences. One of the scaffolds, Lo7_v2_scaffold_617669, whose fragment of the sequence is consistent with the 3581173 DArTSeq marker sequence, does not have a potential candidate gene for this type of dwarfism, but is a good source for searching for a marker associated with the *dw*9 gene.

There is also no clear evidence to determine whether the *dw*9 gene localized on 6RL rye can be matched by another dwarf gene of wheat or barley based on synteny. The genes *Rht*14, *Rht*16, and *Rht*18 [34] are located on 6BS of wheat. Nevertheless, their coincidence with the *dw*9 gene is currently difficult to prove.

Comparing the phenotypic expression of the dwarfing gene *dw*9 with the effects of other recessive genes: *ct*2, *dw*8 and 3 other unknown sources (Milczarski et al. unpublished), it can be concluded that dw9 dwarfism has a different type. This is mainly due to a mutation that significantly increases the number of internodes, causing the dwarf plant to be flexed and unfolded, rather than erected. Additional ears may appear on one stem, but they are very poorly filled with grain. It is not clear whether these disadvantages are only linked to the dwarfing gene or the pleiotropic effect of the gene. The use of this source directly in rye breeding is therefore impossible at this stage, mainly due to the negative effect of this type of dwarfism on yielding traits. Particularly, it is very difficult to maintain the BK-1 line in a vital state. Current backcrossing with Dańkowskie Amber varieties will allow us to answer the question of whether this source of dwarfism can be used in rye breeding in the future.

## Supporting information

S1 Table Biometrical analysis of parental lines, F2, F3 populations, gibberellin test and Dańkowskie Amber x BK-1 cross. (XLSX)

S2 Table Markers segregation in mapping population 541 x Bk-1 on 6R rye chromosome. (XLSX)

S3 Table Alignment analysis of DArTSeq markers sequence to Sc_Lo7_v2_6R scaffolds and annotation. (XLSX)
